# Leaf Extracts from *Dillenia philippinensis* Rolfe Exhibit Cytotoxic Activity to both Drug-Sensitive and Multidrug-Resistant Cancer Cells

**DOI:** 10.31557/APJCP.2019.20.11.3285

**Published:** 2019

**Authors:** Rachelle Anne S Dante, Regina Joyce E Ferrer, Sonia D Jacinto

**Affiliations:** *Institute of Biology, University of the Philippines, Diliman, Quezon City, Philippines. *

**Keywords:** Dillenia philippinensis, natural products, cancer, multidrug resistance, P-glycoprotein

## Abstract

**Background:**

Cancer is one of the leading causes of illness and death worldwide. Only palliative therapeutic options are available for many types of cancers, and most anticancer drugs in clinical use exhibit significant side effects. It is therefore important to develop new anticancer drugs that are more effective and less toxic. In this study, we evaluate the bioactivity of a Philippine endemic plant, “katmon” or *Dillenia philippinensis*, and its potential use in cancer therapy.

**Methods:**

The cytotoxicity of the crude leaf extract, partitions, and isocratic column chromatography fractions of *Dillenia philippinensis* was determined *in vitro* by MTT assay against drug-sensitive cancer cell lines MCF7 (human breast adenocarcinoma) and HCT 116 (human colorectal carcinoma), as well as against moderately multidrug resistant (MDR) cancer cell line HCT-15 (human colorectal carcinoma) and its highly MDR subline HCT-15/Dox. The selectivity of the extract to cancer cells was determined by computing for the selectivity index (SI) with respect to normal mouse embryonic fibroblasts (NIH/3T3) cell line. To check for a possible mechanism for overcoming cancer multiple drug resistance, Calcein-AM assay was performed to assess the activity of the extract against P-glycoprotein-activated efflux pump.

**Results:**

*Dillenia philippinensis* (DP1) fraction from the hexane partition exhibited cytotoxicity (IC_50_< 30 µg/ml) against MCF7, HCT 116, HCT-15, and HCT-15/Dox cells. DP1 also exhibited a moderate level of selectivity against cancer cells over normal cells as supported by the SI computed from the IC_50_ value obtained for the normal cell line. DP1 was able to inhibit P-glycoprotein (P-gp) activity in a dose-dependent manner, suggesting its possible role in targeting cancer cells with overexpressed P-gp.

**Conclusion:**

The present findings thus demonstrate the potential chemotherapeutic properties of *D. philippinensis* which can be promising for future drug development against cancer.

## Introduction

Cancer remains as one of the leading causes of death worldwide, with the latest global statistics of 9.6 million cancer-related deaths and 18.1 million new cases in the year 2018 (Bray et al., 2018). For many tumors, particularly those of advanced stages, therapeutic options are exclusively palliative. Moreover, anticancer drugs available for clinical use commonly have adverse side effects. There is therefore an urgent need to discover and develop new chemotherapeutic agents that are more effective and less toxic. 

One major obstacle in cancer therapy is multiple drug resistance (MDR) in tumors, where cancer cells become insensitive to a wide variety of drugs that could be structurally and functionally unrelated (Housman et al., 2014; Kartal-Yandim, 2016). A common mechanism for both acquired and intrinsic MDR involves the overexpression of ATP-dependent transport protein, P-glycoprotein (P-gp; MDR1; ABCB1) (Szakacs et al., 2006; Housman et al., 2014; Chung et al., 2016). P-gp can actively expel a wide variety of substrate drugs out of the cells, leading to the reduction of the intracellular concentrations of these drugs to sublethal levels (Choi, 2005; Szakacs et al., 2006). Research aimed at overcoming MDR by inhibiting P-gp function has resulted to three generations of inhibitors. However, all P-gp inhibitors developed so far have shown problems such as high toxicity, low specificity, lack of potency, and unknown pharmacokinetic interactions (Palmeira et al., 2012; Kapse-Mistry et al., 2014).

Screening for new drug candidates from natural sources (e.g. plants, microbes, and marine organisms) is a particularly promising approach for overcoming both drug-sensitive and MDR cancer, since chemical scaffolds in natural products have much higher diversity than what can be conceived when synthesizing compounds from scratch. In addition, natural products are generally less toxic and physiologically more easily tolerated inside biological systems (Lahlou, 2013). In this study, we investigated the cytotoxicity of leaf extracts from a Philippine endemic plant, *Dillenia philippinensis* on five cell lines: human breast adenocarcinoma cell line MCF7, human colorectal carcinoma cell line HCT 116, MDR human colorectal adenocarcinoma cell line HCT-15, HCT-15 subline with enhanced multiple drug resistance HCT-15/Dox, and normal mouse embryonic fibroblast cell line NIH/3T3. The cancer cell lines represent two of the most common cancers worldwide: breast cancer which accounts for 11.6% of all recorded cases of cancer in 2018, and colorectal cancer which accounts for 10.2% (Bray et al., 2018). NIH/3T3, on the other hand, was used to assess the toxicity of the extract on non-cancer cells. We also investigated the effect of the most cytotoxic *D. Philippinensis* extract fraction on P-gp function, as a possible mechanism for its anti-MDR cancer activity. 

## Materials and Methods

*Plant Collection and Identification*

The leaves of *Dillenia philippinensis* were collected from the vicinity of the University of the Philippines, Diliman, Quezon City. The identity of the plant sample was authenticated by the staff of Jose Vera Santos Herbarium of the Institute of Biology, UP Diliman where a voucher specimen was deposited under Accession No. 18115.

*Preparation of the crude leaf extract*

The leaves were dried in an oven (Memmert, Germany) at 40°C until crisp and brittle. The dried leaves (midrib removed) were cut into small pieces and homogenized using a blender (Osterizer^®^, USA). The resulting homogenate was soaked in absolute ethanol (RCI Labscan Limited, Thailand) for at least 48 hours before filtration. The filtrate was concentrated in vacuo at 40°C using a rotary evaporator (Heidolph, Germany) and the condensate was air-dried to obtain a concentrated crude ethanolic extract. The extract was stored at 4°C until use.

*Solvent Partitioning*

The crude extract was re-dissolved in 200 mL of 95% ethanol and subjected to successive liquid-liquid extraction using 95% hexane (RCI Labscan Limited, Thailand), ethyl acetate (RCI Labscan Limited, Thailand), and distilled water in order of increasing polarity. The hexane and ethyl acetate partitions were extracted exhaustively and concentrated using a rotary evaporator (Heidolph, Germany) while the aqueous partition was concentrated through lyophilization (Christ, Germany). The concentrated partitions were then stored at 4°C until use.

*Isocratic Silica Gel Column Chromatography*

The most cytotoxic partition as demonstrated through MTT cytotoxicity assay was separated into fractions using silica gel column chromatography. Eight hundred milligrams (800 mg) of the dried partition were added with minimal amounts of 1:1 hexane-ethyl acetate until dissolution. The sample was then loaded into a silica gel 60 (Merck, Germany) column with 1:1 hexane-ethyl acetate as the solvent system. Twenty-milliliter eluents were collected on pre-weighed tubes and were left to air-dry.

*Thin Layer Chromatography*

The fractions per tube were blotted into Thin Layer Chromatography (TLC) silica gel plates (Merck, Germany) using a capillary tube. The plate was then placed in an equilibrated TLC chamber with 1:1 hexane-ethyl acetate as the solvent system. The fractions that showed the same banding profiles were then pooled together resulting in a total of 5 fractions.

*Cell Lines*

The human breast cancer cell line MCF7, human colorectal cancer cell lines HCT 116 and HCT-15, and the mouse embryonic fibroblast cell line NIH/3T3 were purchased from the American Type Culture Collection (ATCC, Manassas, Virginia, USA). An HCT-15 subline with enhanced multiple drug resistance (HCT-15/Dox) was established by continuous exposure of HCT-15 to a sublethal dose of doxorubicin for six months (protocol adapted from Uchiyama-Kokubu and Watanabe, 2001). 

*Cell Culture*

MCF7 cells were cultured in Minimum Essential Medium supplemented with 10% (v/v) FBS, 1% (v/v) 100X antibiotic-antimycotic, and 1% (v/v) 100X Insulin-Transferrin-Selenium. HCT 116 cells were cultured in McCoy’s 5A medium supplemented with 10% FBS, 1% 100X antibiotic-antimycotic, and 1.5% sodium bicarbonate. HCT-15 cells were cultured in RPMI 1640 supplemented with 10% (v/v) Fetal Bovine Serum (FBS) and 1% of Gibco® 100X antibiotic-antimycotic (v/v). HCT-15/Dox was cultured using the same culture medium but with doxorubicin added. NIH/3T3 cells were cultured in Dulbecco’s Modified Eagle Medium (DMEM) supplemented with 10% (v/v) Newborn Calf Serum (NBCS) and 1% (v/v) 100X antibiotic-antimycotic. All culture media components were procured from Gibco™, New York, USA. The cell cultures were incubated under standard culture conditions of 37°C, 5% CO_2_ in a 95% humidified atmosphere.

*(3-(4,5-Dimethylthiazol-2-yl)-2,5-Diphenyltetrazolium Bromide) (MTT) assay*

The assay was performed following the protocol of Mosmann (1983) with minor modifications. A master dilution plate (MDP) of the samples was prepared in a 96-well microtiter plate (Corning, New York, USA). The stock solution for each treatment was made by dissolving 4 mg of the extract in 1 mL Dimethyl Sulfoxide (DMSO; RCI Labscan Limited, Thailand) in a microcentrifuge tube. The stock solutions were used in the treatment of cells together with Doxorubicin (Hospira, Australia) and DMSO as the positive and negative controls, respectively.

To perform the assay, 190 μL cell suspension in culture medium was dispensed per well in a 96-well microtiter plate. The seeding density was 6x10^4^ cells/mL for MCF7 cells and 4x10^4^ cells/mL for HCT 116, HCT-15, HCT-15/Dox, and NIH/3T3 cells. The cells were then treated with the following screening concentrations (serial half-log dilutions) of the extracts 24 hours after seeding: 200, 63.3, 20.0, 6.34, 2.01, 0.64, 0.20, and 0.06 μg/mL. After 72 hours of exposure to the extract, the culture media containing the treatment were discarded and replaced with 20 μL of MTT (5 mg/mL PBS) (AMRESCO, Ohio, USA). The plate was incubated again under standard culture conditions for another 4 hours, then added with 150 μL of DMSO to solubilize the formazan crystals. The amount of formazan generated in each sample was quantified by reading the absorbance of each well at 570 nm using a plate reader (Labexim Products, Austria). The absorbance readings were then used to compute the Inhibition Concentration 50 (IC_50_), the minimum concentration required to kill 50% of the cell population, by plotting the percent inhibition vs. log concentration through non-linear regression analysis using GraphPad Prism version 6.00 (GraphPad Software, California, USA). Based on the values set by the American National Cancer Institute (NCI), an extract is only considered active against cancer cells if the IC_50_ value obtained was less than 30 μg/mL (Suffness and Pezzuto, 1990).

*Calcein AM assay*

Calcein-AM assay was performed to test whether the exposure to DP1, the active fraction, could dampen or inhibit the action of P-gp efflux pumps in the plasma membrane. HCT 116, HCT-15, and HCT-15-Dox cells were seeded in 96-well plates with a seeding density of 5x105 cells/mL in triplicates. After 24 hours of incubation, the culture media were discarded and replaced with 100 µL of fresh RPMI media containing the following treatment concentrations: (a) 5 µg/mL Verapamil (positive control), (b) 0.1x, 1x, and 10x HCT-15 IC_50_ of DP1 (0.913, 9.13, and 91.3 µg/mL, respectively), and (c) DMSO (negative control). Verapamil was used as the positive control as it is an established MDR modulator. The cells were incubated with the treatment for 30 minutes. Afterwards, 50 µL of Calcein was added to each well. The plate was again incubated for another 30 minutes. The fluorescence readings were then measured using Varioskan™ Flash (Thermo Scientific, Massachusetts, USA).

*Statistical Analysis*

Statistical analyses were performed using R (version 3.3.0). ANOVA using the functions “aov” and “anova” followed by Tukey’s multiple comparisons test using the function “TukeyHSD” were used to evaluate treatment effects. Distribution fitting was performed using the package “fitdistrplus” (Delignette-Muller and Dutang, 2015).

## Results

*Cytotoxicity against non-MDR cancer cell lines*

To examine the plant’s bioactivity, *D. philippinensis *crude ethanolic extract was tested against HCT 116 and MCF7 cell lines by MTT assay. Table 1 summarizes the IC_50_ values of the crude extract against the cell lines tested. Following the NCI guideline, the crude extract exhibited an acceptable level of bioactivity (IC_50_< 30 µg/ml), which warrants its further fractionation. Successive liquid-liquid extraction then yielded 3 solvent partitions (hexane, ethyl acetate, and aqueous partition), of which only the hexane partition was included in Table 1 as it was the fraction found cytotoxic. The ethyl acetate and aqueous partitions were not cytotoxic and were no longer pursued in the study. Fractionation of the hexane partition by isocratic silica gel chromatography yielded 5 pooled fractions. Only DP1 fraction was cytotoxic, with mean IC_50_ values of 15.59±0.22 µg/mL and 14.23±0.65 µg/mL against MCF7 and HCT 116 cells, respectively (Table 1). The remaining fractions were found to be non-cytotoxic against the cancer cells tested.

*Cytotoxicity against MDR cancer cell lines*

**Figure 1:**
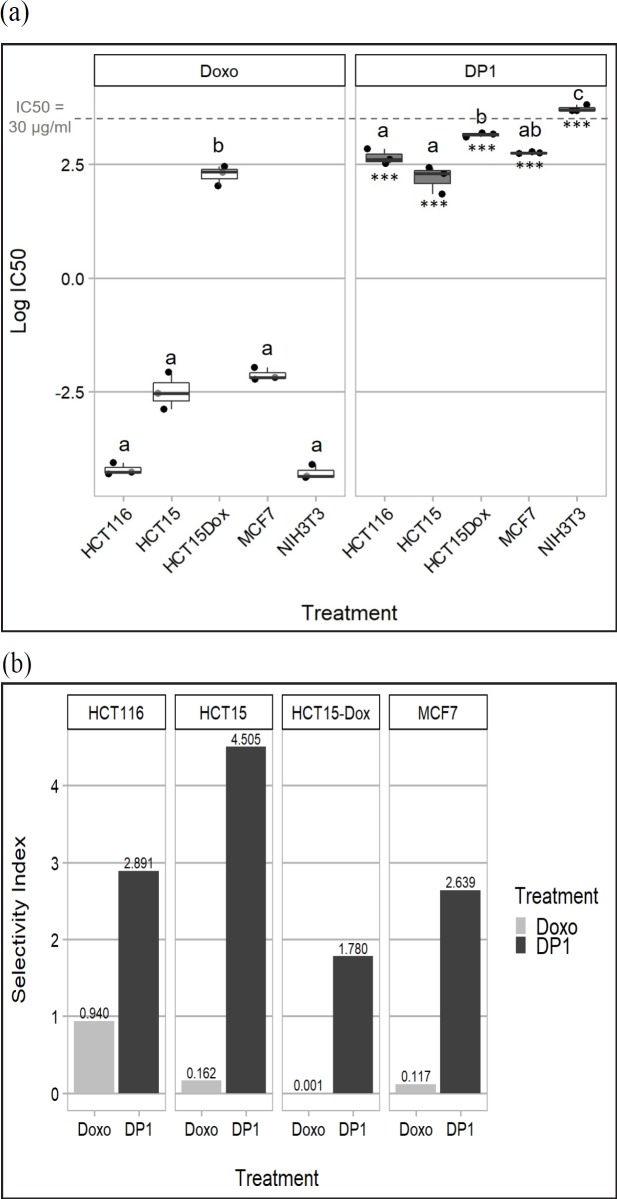
Mean IC_50_ values of DP1 Fraction Against HCT 116, HCT-15, HCT-15/Dox, and MCF7 Cancer Cell Lines and NIH/3T3 Non-Cancer Cell Line (a) and the corresponding selectivity index (SI) values (b). Doxorubicin serves as the positive control. Extracts with SI values >3 are considered as highly selective to cancer cells (Prayong et al., 2008; Sufian et al., 2013). Data represent three trials performed in triplicate wells. Letters signify groupings based on two-way ANOVA with Tukey’s multiple comparisons test that compared IC_50_ across cell lines for each extract. *** signify significant difference from Doxorubicin’s IC_50_ (p < 0.001, two-way ANOVA with Tukey’s multiple comparisons test)

To determine whether *D. philippinensis* is also capable of targeting MDR cells, the crude extract, partitions, and isocratic fractions were then tested against HCT-15 cells, a cell line exhibiting MDR phenotype and is characterized by high P-gp expression and higher resistance to MDR-associated drugs than to non-MDR drugs (Wu et al., 1992; Izquierdo et al., 1996; Uchiyama-Kokubu and Watanabe, 2001). Results showed that comparable to the drug-sensitive cell lines, the crude extract, hexane partition, and DP1 fraction maintained their cytotoxic activity against HCT-15 cells (Table 1; Figure 1-a), with DP1 having a mean IC_50_ value of 9.13±1.45 µg/mL. DP1 was also tested against a more resistant, Doxorubicin-induced HCT-15 (HCT-15/Dox) subline to further assess its potential in targeting resistant cancer cells. The results showed that DP1 still exhibited an acceptable level of cytotoxic activity, comparably less than that of the parent cell line (Figure 1-a), with an IC_50_ value of 23.11±0.67 µg/mL (Table 1).

*Therapeutic selectivity of DP1 fraction to cancer cells*

To determine the effect of DP1 on normal cell viability, it was tested against NIH/3T3 cells, a non-cancer mouse embryonic fibroblast cell line, using MTT assay. The selectivity index (SI) of DP1 to cancer cells was also determined using the following formula:

$\mathrm{SI}=\frac{{\mathrm{IC}}_{50}\mathrm{against NIH}/3\mathrm{Ts}}{{\mathrm{IC}}_{50}\mathrm{against cancer cell line}}$

Treatments with high SI values are projected to have fewer adverse effects. Figure 1-b shows the selectivity indices of control drug doxorubicin and DP1 fraction on cancer cell lines. An extract with a selectivity index value higher than 3 was considered as highly selective to cancer cells (Prayong et al., 2008; Sufian et al., 2013). Results showed that with respect to the NIH/3T3 cell line, DP1 exhibited high selectivity towards HCT-15 cells, moderate selectivity towards HCT 116 and MCF7 cells, and low selectivity towards HCT-15/Dox cells (Figure 1-b). DP1 also had higher selectivity indices than doxorubicin in all four cancer cell lines tested (Figure 1-b). This validated by the statistical analysis of IC_50_ data, which showed that DP1 was significantly less cytotoxic against NIH/3T3 compared to the four cancer cell lines tested, while doxorubicin was equally cytotoxic against normal cells and non-MDR cancer cells (Figure 1-a).

*Calcein AM assay*

**Figure 2:**
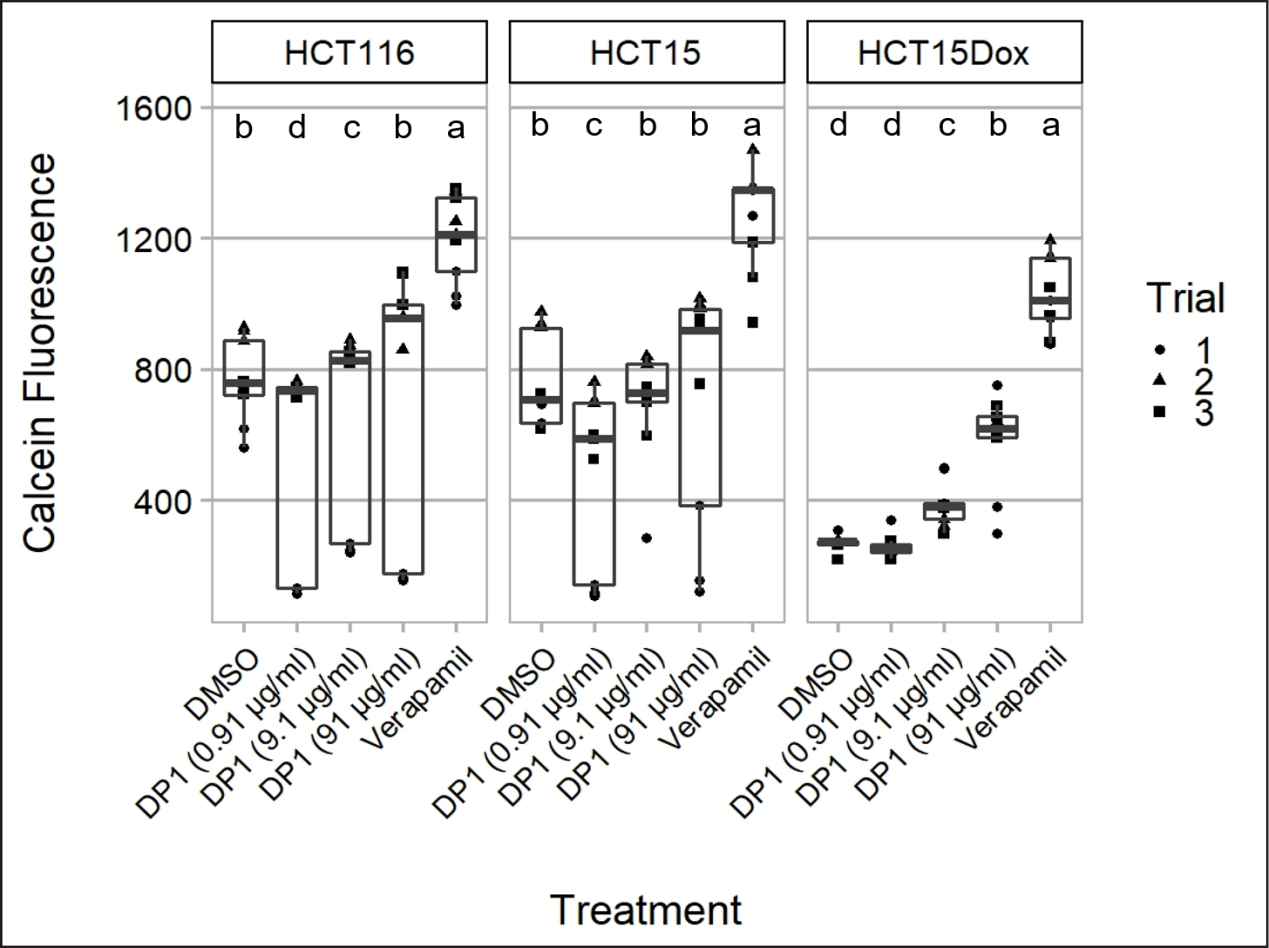
Mean Calcein Fluorescence Values of Verapamil (Positive Control), DMSO (Negative Control), and Different Concentrations of DP1 Extract against HCT116, HCT-15, and HCT-15/Dox Cells. The data represent mean ± SD of three independent trials. Letters signify groupings based on one-way ANOVA with Tukey’s multiple comparisons test that compared calcein fluorescence across treatments for each cell line

Calcein AM is an established protocol used to quantify MDR efflux activity. This assay was then performed to determine if DP1 can target resistant cells by modulating P-gp function. Three 10-fold concentrations of DP1 were tested for their P-gp inhibitory activity in three cell lines, HCT 116, HCT-15, and HCT-15/Dox cell lines, which were differentiated by their increasing levels (low, moderate, and high) of inherent P-gp expression, respectively (Ferrer, 2018). Results showed that DP1 elicited a dose-dependent response on P-gp inhibition in the three cell lines tested, with increasing calcein retention as the concentration of DP1 increases (Figure 2). For the drug resistant cell line HCT-15/Dox, DP1 significantly inhibited the P-gp efflux pumps in a dose-dependent manner after 30 mins. of treatment exposure, but at less efficacy compared to the positive control, Verapamil (Figure 2). Similarly, the trend observed in HCT 116 and HCT-15 cells was dose-dependent, although the observed activity was not significant as compared to the negative control (Figure 2). For all three cell lines, the lowest concentration tested (0.91 μg/ml) decreased calcein fluorescence relative to the negative control (significantly in HCT116 and HCT-15), which may indicate stimulation of P-gp activity.

## Discussion

Plants contribute significantly to the roster of anticancer drugs used in medical care worldwide. Some of the notable plant-derived drugs include paclitaxel and vinca alkaloids: vincristine and vinblastine (Singh et al., 2016). From the vast biodiversity of flora in the Philippines, *Dillenia philippinensis* (katmon) was investigated as it has shown potential in the preliminary screening of Philippine endemic and indigenous plants. The results of the screening, together with chemical studies by Ragasa et al., (2009) and Macahig et al., (2011), demonstrated that *D. philippinensis* leaves harbor potentially active anticancer compounds. From the bioactivity-guided partitioning and fractionation of the crude extract, the isocratic column chromatography fraction 1 (DP1) from the hexane partition showed potent cytotoxicity against both drug-sensitive and resistant colorectal carcinoma cell lines.

DP1 was cytotoxic (IC_50_< 30 μg/ml) against breast cancer cell line MCF7, and three colon cancer cell lines of varying resistance: HCT 116 (non-MDR), HCT-15 (moderately MDR; Wu et al., 1992; Izquierdo et al., 1996; Uchiyama-Kokubu and Watanabe, 2001) and HCT-15-Dox (highly MDR; Suanes, 2019). Although DP1 was significantly less potent than the control drug Doxorubicin for all four cell lines, it is interesting to note that DP1’s cytotoxic activity was not diminished in HCT-15 (compared to HCT 116) and was only reduced by 1.6- fold in HCT-15/Dox. Doxorubicin, on the other hand, is a substrate of P-gp causing it to be 9-fold less potent against HCT-15 and almost 1000-fold less potent against HCT-15-Dox compared to HCT 116 cells. In a similar way, many of the widely used chemotherapeutic agents are also P-gp substrates and are easily transported outside of the cell, thereby limiting their bioavailability and effectivity (Choi, 2005; Szakacs et al., 2006).

DP1 elicited a dose-dependent reduction of P-gp efflux activity in the highly resistant HCT-15/Dox cells after 30 minutes of treatment. Based on the short period of time required to elicit the inhibitory effect, DP1 likely inhibits P-gp by directly binding to it. Inhibition of P-gp function was significant at higher doses, allowing it to maintain lethal concentrations inside the resistant cells. However, this inhibitory activity is lost at lower concentrations; more specifically, DP1 stimulated P-gp function at low concentrations in the three colorectal cancer cell lines (albeit not significantly in HCT-15/Dox). This biphasic effect - stimulatory at low concentrations and inhibitory at high concentrations - is commonly observed in P-gp inhibitors (Calabrese, 2008). On the other hand, although the P-gp inhibitory effect of DP1 was not significant in HCT 116 and HCT-15 cells that have lower P-gp expression (Ferrer, 2018), the biphasic trend of the response that was observed in HCT-15/Dox was also observed in these cell lines. The lack of statistical significance is possibly due to the higher standard deviation in the data for these two cell lines. 

Results of the MTT assay on the non-cancer mouse fibroblast cell line NIH/3T3 reveal that DP1 selectively acts against cancer cells. The level of therapeutic selectivity however, notably varied depending on the cell line. Computed selectivity indices indicate high selectivity for HCT-15, moderate selectivity for HCT 116 and MCF7, and low selectivity for HCT-15/Dox. Nevertheless, DP1 exhibited better selectivity than the control drug Doxorubicin across all cell lines and was significantly less cytotoxic against NIH/3T3 than Doxorubicin (p = 0.000). Despite these promising results, further studies are still necessary to ascertain the safety of DP1, especially inside biological systems. 

Based on a preliminary study, phytochemical screening of DP1 revealed the presence of flavonoids, which are suspected to be the class of compounds responsible for the observed bioactivity. Flavonoids, together with terpenoids, are the major classes of compounds widely reported in many *Dillenia* species (Lima et al., 2014; Sabandar et al., 2017). Flavonoids are recognized as a rich source of compounds with anticancer properties. A number of flavonoids, including flavopiridol, genistein and quercetin, have entered late phase clinical trials for several chemotherapeutic indications (Ravishankar et al., 2013). Flavonoids have diverse structural patterns leading to a wide variety of biological effects including induction of apoptosis, cell cycle arrest and disruption of mitotic spindle formation (Ravishankar et al., 2013). They are also well-known modulators of P-gp mediated drug resistance that act by directly interacting with P-gp (Ravishankar et al., 2013).

Summing all up, DP1 from *D. philippinensis* offered an alternative approach in addressing cancer, which involves chemotherapeutic modalities against both drug-sensitive and resistant cancer cells. While many studies are still needed to unveil its full potential and safety profiles, the cytotoxic activity and generally moderate selectivity of DP1 *in vitro* make it a good candidate for further exploration as a potential therapeutic agent against cancer.

**Table 1:** Mean IC_50_ Values (µg/mL) of *D. philippinensis* Leaf Extracts Against MCF7, HCT 116, HCT-15, and HCT-15/Dox Cancer Cell Lines and the NIH/3T3 Non-Cancer Cell Line as Determined by MTT Assay. Values are means of at least three independent experiments

Treatment	Mean IC_50_ ± SEM per cell line				
	MCF7	HCT 116	HCT-15	HCT-15/Dox	NIH3T3
Doxorubicin	0.12±0.01	0.01±0.001	0.09±0.02	9.76±1.18	0.01±0.001
DP crude	11.92±1.91	17.97±1.32	15.39±0.65		
DP hexane	14.69±1.76	14.98±1.13	11.25±3.31		
DP1 fraction	15.59±0.22	14.23±0.65	9.13±1.45	23.11±0.67	41.14±1.77
